# When Friendship Turns Sour: Effective Communication Between Mitochondria and Intracellular Organelles in Parkinson's Disease

**DOI:** 10.3389/fcell.2020.607392

**Published:** 2020-11-30

**Authors:** Tsu-Kung Lin, Kai-Jung Lin, Kai-Lieh Lin, Chia-Wei Liou, Shang-Der Chen, Yao-Chung Chuang, Pei-Wen Wang, Jiin-Haur Chuang, Tzu-Jou Wang

**Affiliations:** ^1^Center for Mitochondrial Research and Medicine, Kaohsiung Chang Gung Memorial Hospital and Chang Gung University College of Medicine, Kaohsiung, Taiwan; ^2^Department of Neurology, Kaohsiung Chang Gung Memorial Hospital and Chang Gung University College of Medicine, Kaohsiung, Taiwan; ^3^Center of Parkinson's Disease, Kaohsiung Chang Gung Memorial Hospital and Chang Gung University College of Medicine, Kaohsiung, Taiwan; ^4^Department of Anesthesiology, Kaohsiung Chang Gung Memorial Hospital and Chang Gung University College of Medicine, Kaohsiung, Taiwan; ^5^Department of Metabolism, Kaohsiung Chang Gung Memorial Hospital and Chang Gung University College of Medicine, Kaohsiung, Taiwan; ^6^Department of Pediatric Surgery, Kaohsiung Chang Gung Memorial Hospital and Chang Gung University College of Medicine, Kaohsiung, Taiwan; ^7^Department of Pediatric, Kaohsiung Chang Gung Memorial Hospital and Chang Gung University College of Medicine, Kaohsiung, Taiwan

**Keywords:** Parkinson's disease, mitochondria, interorganelle communication, mitophagy, lysosome, endoplasmic reticulum, peroxisome, golgi apparatus

## Abstract

Parkinson's disease (PD) is a complex neurodegenerative disease with pathological hallmarks including progressive neuronal loss from the substantia nigra pars compacta and α-synuclein intraneuronal inclusions, known as Lewy bodies. Although the etiology of PD remains elusive, mitochondrial damage has been established to take center stage in the pathogenesis of PD. Mitochondria are critical to cellular energy production, metabolism, homeostasis, and stress responses; the association with PD emphasizes the importance of maintenance of mitochondrial network integrity. To accomplish the pleiotropic functions, mitochondria are dynamic not only within their own network but also in orchestrated coordination with other organelles in the cellular community. Through physical contact sites, signal transduction, and vesicle transport, mitochondria and intracellular organelles achieve the goals of calcium homeostasis, redox homeostasis, protein homeostasis, autophagy, and apoptosis. Herein, we review the finely tuned interactions between mitochondria and surrounding intracellular organelles, with focus on the nucleus, endoplasmic reticulum, Golgi apparatus, peroxisomes, and lysosomes. Participants that may contribute to the pathogenic mechanisms of PD will be highlighted in this review.

## Introduction

Parkinson's disease (PD) is the second most common neurodegenerative disease after Alzheimer's disease (Poewe et al., 2017), affecting ~1% of the population over the age of 60, resulting in a significantly shorter life span for PD patients (Abbas et al., 2018). This progressive neurodegenerative disease has two histopathological hallmarks: loss of dopaminergic neurons in the substantia nigra pars compacta and the presence of intraneuronal α-synuclein (α-syn) protein inclusions called Lewy bodies (Klemann et al., 2017). Decreased dopamine secretion leads to cardinal PD motor phenotypes including resting tremor, bradykinesia, rigidity, and postural instability. These may be preceded by a prodromal phase of up to decades, characterized by specific non-motor symptoms such as rapid eye movement (REM) sleep behavior disorder, depression, constipation, hyposmia, and cognitive impairment (Chaudhuri et al., 2006). Although tremendous progress has been made in the pharmaceutical treatment of PD, the exact causes of PD remain unclear, although important risk factors have been identified, which include aging, genetics, and environmental factors (Poewe et al., 2017; Abbas et al., 2018). Most PD cases are sporadic, while only 5–10% are of the familial form. However, recent genetic discoveries have provided us with valuable insight into the molecular pathways involved in the neurodegenerative process of PD. Several biological processes, including mitochondrial dysfunction, α-syn aggregation, oxidative stress, defective endo-lysosomal functioning, and immune response activation, have been suggested to contribute to the pathogenesis of PD, and these molecular pathways appear to overlap at multiple points (Cipolla and Lodhi, 2017; Grunewald et al., 2019; Nguyen et al., 2019).

An important pathological characteristic of PD is the abnormal accumulation of the misfolded protein, α-syn (encoded by the SNCA gene). Being a major component of Lewy bodies in PD patients, α-syn aggregates have been suggested to play a critical role in PD pathogenesis. Although the physiological role of α-syn awaits elucidation, the detrimental outcome of α-syn oligomers and its aggregates has been the focus of extensive study. In pathological conditions, α-syn can form insoluble fibrils through oligomerization (Hijaz and Volpicelli-Daley, 2020). The α-syn oligomer induces overproduction of ROS due to mitochondrial respiratory complex I inhibition, leading to mitochondrial dysfunction. The link between mitochondrial dysfunction and PD dates back to 1982 when seven adolescents developed parkinsonism shortly after injection of synthetic heroin containing byproduct 1-methyl-4-phenyl-1,2,3,6-tetrahydropyridine (MPTP), a specific mitochondrial respiratory chain complex I inhibitor (Nonnekes et al., 2018). Since then, a myriad of studies have demonstrated the causal role of mitochondrial dysfunction in PD, including many established PD models inhibiting respiratory complex I such as MPTP, the pesticide rotenone, and herbicide paraquat (Blesa and Przedborski, 2014). More recent genetic discoveries have underlined the importance of mitochondrial integrity in the neurodegenerative process of PD, as many familial PD genes encode proteins essential in mitochondrial homeostasis. Genome-wide association studies (GWAS) and transcriptome-wide association study (TWAS) have, respectively, identified 41 and 66 susceptible loci associated with PD, including autosomal recessive PD genes *PRKN/PARK2, phosphatase and tensin homolog (PTEN)-induced putative kinase 1 (PINK1)/PARK6, Daisuke-Junko-1 (DJ-1)/PARK7, ATPase type13A2 (ATP13A2)/PARK9*, and *F-box only protein 7 (FBXO7)/PARK15*, and autosomal dominant PD genes *SNCA/PARK1, leucine-rich repeat kinase 2 (LRRK2)/PARK8*, and *vacuolar protein sorting 35 (VPS35)/PARK17* (Li et al., 2019b). All of these genes have been shown to affect mitochondrial biogenesis, morphology, trafficking, and elimination (Chang et al., 2017; Li et al., 2019b), further supporting the role of mitochondrial dysfunction in the pathophysiology of both sporadic and familial forms of PD.

In order to exert their complex activities, mitochondria must actively signal and interact with other subcellular compartments. This coordination between mitochondria and different organelles requires frequent communication via direct contact or indirect interorganelle signaling (Giacomello et al., 2020). Recent evidence has shown how interorganelle communication impacts mitochondrial and cellular functions from susceptible PD genes. Close communication between the nucleus and mitochondria through anterograde and retrograde signaling pathways plays a pivotal factor in maintaining mitochondrial integrity in response to cytosolic/mitochondrial stress for cellular survival (Schumacher and Vijg, 2019). Furthermore, the interactions between mitochondrial and endoplasmic reticulum (ER) are essential for normal mitochondrial functioning. These two organelles form tight contact sites for molecular transfers, which includes Ca^2+^ and metabolites such as lipids (Lee and Min, 2018). Silencing of various PD genes causes disruption of mitochondria/ER contact sites, increased ER stress, mitochondrial fragmentation, inhibition of mitophagy, and neuronal death (Tapias, 2019). The Golgi apparatus has also been observed to interact with mitochondria and ER for regulating Ca^2+^ homeostasis, and mutant α-syn has been shown to disturb ER–Golgi trafficking. The susceptible-PD-gene-encoded trafficking protein VPS 35 has been shown to be localized at the Golgi apparatus, suggesting their involvement in mitochondria-related protein transport (Ebanks et al., 2019). Recently, mounting evidence has indicated the interaction between peroxisomes and mitochondria. Both organelles play important roles in lipid β-oxidation metabolism, and both play a redox scavenging role to maintain cellular reactive oxygen species (ROS) homeostasis (Uzor et al., 2020). Interdependence between the two organelles has been noted, as dysfunction in one leads to dysfunction in the other. Evidence suggests that both peroxisomal and mitochondrial dysfunction contribute to organismal aging and is involved in neurodegenerative diseases (Cipolla and Lodhi, 2017). Moreover, since the early 2000's, evidence has demonstrated that lysosomal and mitochondrial dysfunctions in PD are inseparable, as lysosomal degradation of damaged mitochondria is crucial in mitochondrial quality control. Further, studies have also demonstrated the reciprocal relationship between mitochondria and lysosomes (Deus et al., 2020). Thus, interorganelle communication, with mitochondria as a hub, is important in intracellular function, and when friends become foes, pathophysiological connections to diseases ensue. In the following sections, we will review the role of mitochondria in cellular functions and the distinct interactions of the interorganelle network and discuss how this crosstalk may impact PD pathogenesis.

## Mitochondrial Biology

Mitochondria have been a major focus for research endeavors for over 30 years due to their involvement in cellular ROS production and the induction of apoptosis (Pfanner et al., 2019). This is in part due to a growing acknowledgment of the role of mitochondria in multiple functions other than energy production, including Ca^2+^ homeostasis, generation of ROS, regulation of apoptosis, activation of endoplasmic reticulum (ER)-stress response, and other consequences of mitochondrial dysfunction (Giorgi et al., 2018; Spinelli and Haigis, 2018). Mitochondria are also implicated in many neurodegenerative diseases, such as Alzheimer's and Parkinson's disease, as well as in the aging process. The organelle is under the control of both mitochondrial and nuclear genomes, leading to a potential mosaic of pathogenic mutations (Quirós et al., 2016). A majority of mitochondrial proteins are encoded by nuclear genes and follow the usual pattern of Mendelian inheritance. A few [2 ribosomal RNAs, 22 tRNAs, and 12 subunits of the electron transport chain (ETC)] are encoded by mitochondrial DNA (mtDNA), a circular genome within the mitochondria that is 13,794 nucleotides in length (Giacomello et al., 2020).

The mitochondria contain two membranes, the mitochondrial outer membrane (MOM) and the mitochondrial inner membrane (MIM), with the intermembrane space (IMS) between the two and a central mitochondrial matrix. The MIM is highly folded, creating deep invaginations called cristae, which provide a large surface area harboring ETC complexes I–IV and F_1_F_0_-ATP synthase for ATP production (Capaldi and Aggeler, 2002). Reducing equivalents (NADH and FADH_2_) generated from the tricarboxylic acid (TCA) cycle in the matrix pass electrons on to the ETC in the MIM, with O_2_ serving as the terminal electron acceptor to form water. The ETC enzyme complexes I–IV use energy generated from the redox reactions to pump protons across the MIM into the IMS. Complexes I and IV are proton pumps, while complex III is not considered a true pump but rather a Mitchellian proton-loop machine (Stuchebrukhov, 2018). Impermeable to protons, the MIM acts as a functional barrier and establishes a proton-motive force comprised of electrical and chemical potentials across the MIM, termed mitochondrial membrane potential (Δψm). This proton gradient generated from electron transportation (oxidation) then drives protons across the MIM through the F_1_F_0_-ATP synthase (complex V) and ultimately phosphorylate adenosine diphosphate (ADP) into ATP (phosphorylation), called oxidative-phosphorylation (OXPHOS) coupling (Lu, 2011). Δψm is also a crucial indicator for mitochondrial health and, if dissipated, may signal the cell to perform various stress responses or even mitochondrial mediated apoptosis (Mitchell, 2011). However, ROS are generated from up to 12 different enzymes associated with nutrient metabolism and OXPHOS, including several flavoproteins and respiratory complexes I–III (Gorrini et al., 2013; Mailloux, 2020). ROS are highly reactive and can imbalance cellular reduction–oxidization (redox) and readily oxidize proteins, lipids, carbohydrates, DNA, and RNA causing oxidative damage if in excess. To protect organelles from ROS oxidative damage, mitochondria develop their own antioxidative system, such as the protective antioxidative manganese superoxide dismutase 2 (SOD2) enzymes and the glutathione/glutathione peroxidase/glutathione reductase axis (Beer et al., 2004; Lin et al., 2018; Mailloux, 2020) With its close association with the OXPHOS system, the mitochondrial genome is subject to ROS assault (Saki and Prakash, 2017). Thus, mitochondria are heavily dependent on antioxidative enzymes encoded by the nuclear genome (Kazak et al., 2012). Both mtDNA mutation and nuclear DNA repair defects are considered cellular mechanisms of aging, and a recent study has also found that mtDNA can destabilize nuclear genome maintenance (Hamalainen et al., 2019). In the normal aging process, mtDNA mutations cause respiratory chain deficiencies and deficient protein homeostasis (proteostasis) (Schumacher and Vijg, 2019). These findings are observed in the PD brain in association with α-syn aggregation, especially in the substanstia nigra (Dolle et al., 2016; Rango and Bresolin, 2018).

Mitochondria also play major roles in the decision regarding cell fate through intrinsic mitochondrial apoptotic pathways. Regulating both cellular and mitochondrial Ca^2+^ distribution, mitochondria sense alterations of intracellular Ca^2+^ homeostasis. Uncontrolled mitochondrial Ca^2+^ overloading and ROS overproduction induce cell death by triggering mitochondrial permeability transition pore (mPTP) opening (Feno et al., 2019). Sustained mPTP opening leads to the collapse of Δψm, swelling of the mitochondria, cytochrome c and proapoptotic mediators release from the mitochondrial intermembrane space to the cytosol, and eventual cellular apoptosis. The release of cytochrome c from the mitochondria to the cytosol activates downstream proapoptotic mediators including apoptotic peptidase-activating factor 1 (APAF1), pro-caspase-9, and apoptosome-dependent activation of caspase 3, caspase 6, and caspase 7, the executioners of apoptosis. Mitochondrial Ca^2+^ homeostasis is regulated by electrogenic Ca^2+^uptake [via mitochondrial Ca^2+^ uniporter (MCU)] and efflux (in excitable cells via Na^+^/Ca^2+^ exchanger NCLX) (Dupont and Combettes, 2016). The mitochondria are strategically placed throughout the cell, and mitochondrial Ca^2+^ influx stimulates Ca^2+^-dependent dehydrogenases, which use NADH/FADH2 and activate the electron. NCLX inhibition has been indicated in a familial form of Parkinson's disease, in which PINK-1 deficiency leads to a delayed Ca^2+^ efflux and mitochondrial Ca^2+^ overload in response to physiological Ca^2+^ stimulation (Glancy and Balaban, 2012).

With an array of mitochondrial functions, the maintenance of a healthy pool of mitochondria is critical, and this versatile organelle has developed protective measures for dysfunctional mitochondria through quality control (Eisner et al., 2018). These processes include mitochondrial dynamics of fusion, fission, trafficking, and clearance through mitophagy. Morphological changes in the mitochondrial network to fusion provides macromolecular exchange between the neighboring mitochondria and complements mtDNA. Fusion processes restore functional proteins and non-damaged mtDNA to dysfunctional mitochondria, decreasing the occurrence of mitophagy. Major fusion machinery includes three GTPases: the mitofusins 1 and 2 (Mfn1 and Mfn2) mediate MOM fusion, and the optic dominant atrophy (OPA1) mediates MIM fusion (Picca et al., 2018). The fission of the mitochondria segregates dysfunctional parts of the mitochondria, and these fragmented mitochondria can subsequently be degraded through mitophagy. This fragmented morphology allows for more efficient engulfment by autolysosome machinery, while the interconnected tubular mitochondrial morphology is protective. Master mitochondrial fission proteins include the dynamin-related GTPase protein 1 (Drp1), the mammalian Drp1 homolog dynamin-like protein 1 (DLP1), and mitochondrial fission 1 protein (Fis1) (Fonseca et al., 2019). As defective mitochondria can be detrimental to the cell, the elimination of the entire or partial mitochondria is essential and mainly mediated via three pathways. First, mitophagy via the selective autophagy lysosomal pathway is initiated to remove the entire organelle either in dysfunction or as superfluous. Second, mitochondrial-derived vesicles (MDVs) are released from mitochondria to target selectively chosen damaged mitochondrial portions to the lysosome for degradation in a mitophagy-independent manner. Third, the proteolytic control of mitochondrial protein misfolding facilitates clearance of misfolded proteins to allow for replacement with newly synthesized polypeptides (Pickles et al., 2018). There are a number of mitophagy mechanisms, and the most well-known is mediated by the familial PD gene-encoded proteins, PINK1 and parkin (Palikaras et al., 2018). PINK1 is localized to the MOM, and in a normal functioning mitochondria, PINK1 is rapidly imported into mitochondria to be cleaved by proteases and further degraded by the ubiquitin proteasome system. During mitochondrial dysfunction, Δψm is dissipated, protein import is hindered, and PINK1 are stabilized on the MOM (Gladkova et al., 2018). Auto-activated on MOM, PINK1 recruits parkin to MOM and phosphorylate/activates parkin. Parkin then works to ubiquitinate MOM proteins, while PINK1 phosphorylates the ubiquitin (Ub), and poly-Ub chains are formed. The poly-Ub chains mark the damaged mitochondria for degradation and are recognized by the autophagic adaptor proteins (p62, OPTN, NDP52), which bind with microtubule-associated protein 1A/1B-light chain 3 (LC3) on the growing phagophore membrane through LC3-interacting region (LIR) (Runwal et al., 2019). The strip of phagophore encircles the damaged mitochondria and forms a double-membraned autophagosome, which further fuses with lysosomes to facilitate defective mitochondria elimination (Dikic and Elazar, 2018). Another mitochondrial dynamic characteristic is the trafficking of mitochondria, which is essential for energy and Ca^2+^ distribution and is dependent on mitochondrial Rho (Miro) GTPases. Miro 1 and 2 localize to MOM and form complexes with the TRAK adaptors and dynein or kinesin motors to transport mitochondria along microtubules (Modi et al., 2019).

**Figure 1:**
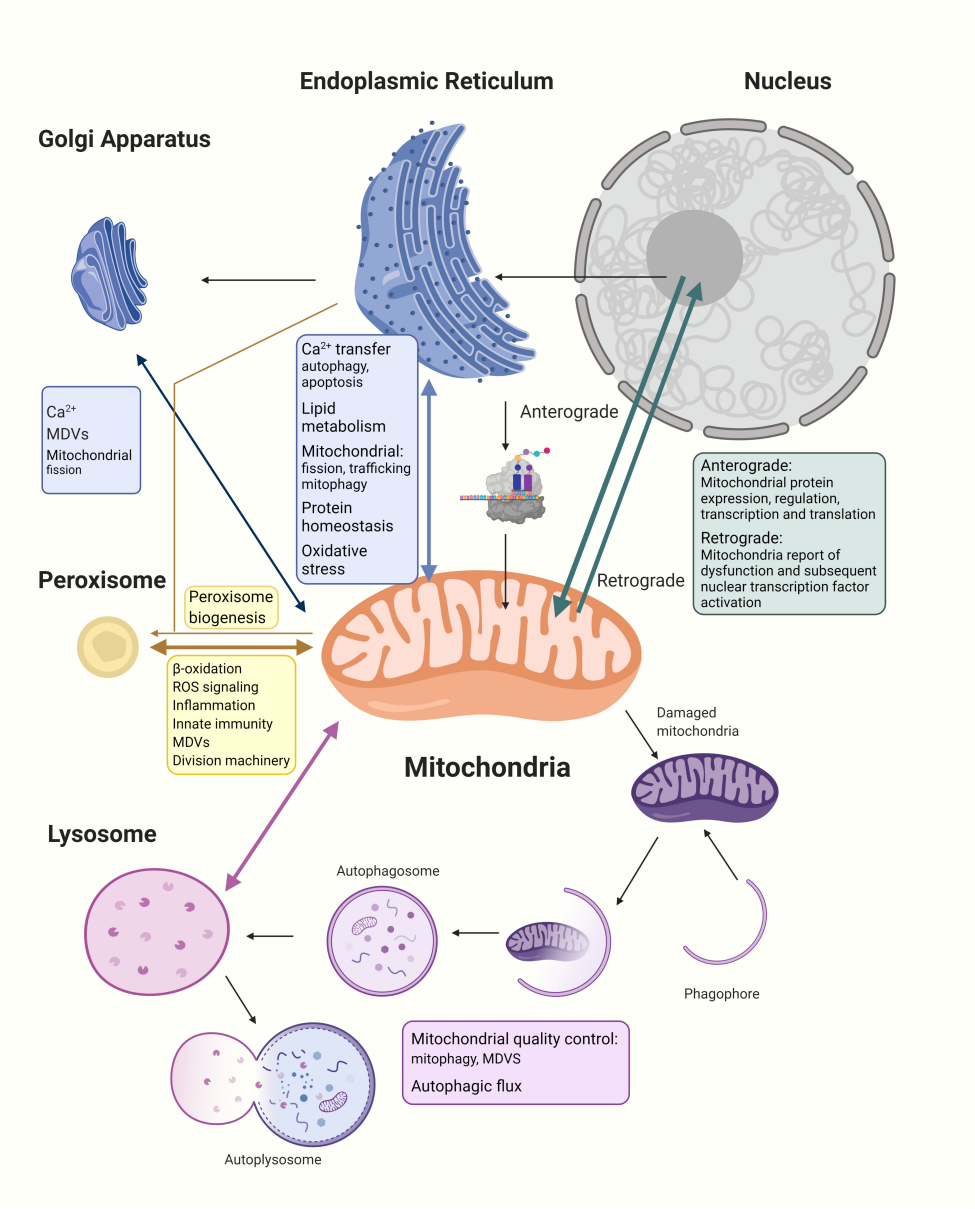
The mitochondria establish networks of communication with other organelles within the cellular community. (1) The nucleus regulates expression of all mitochondrial proteins, including the 99% nucleus-encoded and the remaining mitochondria-encoded 13 peptides manufactured within the mitochondria. Anterograde signaling are nucleus-controlled genetic expressions of mitochondrial proteins through transcription and nuclear factors to regulate mitochondrial biogenesis, while retrograde signaling pathways allow the stressed organelle to report and regulate nuclear gene transcription to decrease the need for energy and allow repairing of dysfunctional mitochondria. (2) In communicating with the ER, Ca^2+^ and lipids are transferred between the mitochondria and ER, and the MERCs regulate mitochondrial fission, trafficking, and mitophagy; moreover, the two organelles provide feedback to the nucleus about protein homeostasis and oxidative stress. (3) The Golgi apparatus has been shown to participate in intracellular Ca^2+^ transport with mitochondria and ER as well as share cargo proteins with mitochondria possibly to do with MDVs trafficking. There has also been evidence of Golgi participation in late-stage mitochondrial fission. (4) The mitochondria and peroxisomes cooperate in lipid oxidation, signaling of reactive oxidative species, regulation of inflammation, and innate immunity. Division machinery of both organelles are overlapped. Mitochondria are also discovered to be involved in peroxisome biogenesis. (5) Lysosomes govern important parts of mitochondrial quality control including whole mitochondrial degradation through mitophagy and the degradation of mitochondrial proteins through MDVs. Dysfunctional mitochondria are shown to decrease the autophagic flux and lysosome health, while lysosome dysfunction also affects mitochondria morphology and function. The interdependency between different organelles and mitochondria highlight the importance of intricate communications and balance within the cellular community for the maintenance of normal functioning cells.

Therefore, the mitochondria are cellular energy providers, damage sensors, Ca^2+^ regulators, and cell death initiators. The versatility of mitochondrial functions relies upon effective communication between the organelle and the entire cellular community. Below, we investigate mitochondrial interactions with other cellular organelles (*Abbreviations* and Figure 1).

## Mitochondrial Communication With the Nucleus

As it has been hypothesized that mitochondria evolved from an independent α-proteobacteria (Degli Esposti, 2014), they retained prokaryotic characteristics such as the double-membraned structure, the ability to aerobically synthesize ATP, and the possession of their own DNA. In evolution, autonomy was lost, and mitochondria became dependent on their host cells; thus, mitochondrial biogenesis and bioenergetics became nuclear regulated (Cherry and Piantadosi, 2015). As has been succinctly reviewed by Quirós et al. (2016), the communication between the mitochondria and the nucleus is a requirement not only to coordinate mitochondrial protein synthesis during biogenesis but also to communicate eventual mitochondrial malfunctions, triggering compensatory responses in the nucleus. The majority of mitochondrial proteins required in mitochondrial biogenesis is encoded by nuclear genes, synthesized on cytosolic ribosomes, directed to mitochondria via targeting signals, and imported into the mitochondria through mitochondrial membrane translocase complexes (Pfanner et al., 2019). Therefore, mitochondrial biogenesis is under dual genetic control from both the nucleus and mitochondria; while all factors that activate mitochondria transcription and translation are encoded in the nucleus, it is mainly the nuclear respiratory factor 1 (NRF1) and GA-binding protein-α (GABPα; also known as NRF2α) that regulate mitochondrial transcription and translation in both the nucleus and mitochondria (Gureev et al., 2019). Additionally, there are nuclear receptors such as the peroxisome-proliferator-activated receptors (PPARs) and estrogen-related receptors (ERRs) that activate the expression of nuclear-encoded mitochondrial proteins. Coactivators for stimulating mitochondrial biogenesis include the PPAR-γ coactivator (PGC) family, which are master regulators of mitochondrial biogenesis and play central roles in the coordination and driving of energy metabolism, fatty acid oxidation, gluconeogenesis, peroxisomal remodeling, and oxidative phosphorylation (Scarpulla et al., 2012). Among them, PGC-1α integrates and coordinates the activity of multiple transcription factors, including NRFs, ERRs, and PPARs, and mitochondrial transcription factor A (TFAM), which are all involved in mitochondrial biogenesis (Gleyzer and Scarpulla, 2011). Additionally, NRF1 integrates the mtDNA gene expression through direct control of the expression of important mitochondrial transcription machinery proteins: the mitochondrial RNA polymerase (POLRMT), TFAM, transcription specificity factors (TFB1M and TFB2M), and transcription termination factor (mTERF) (28). These machineries are made in the cytosol, shipped to the mitochondria, and aid in mtDNA transcription, maintenance, replication, and repair.

A delicate balance between nuclear- and mitochondria-encoded mitochondrial proteins is under continuous monitoring for organelle health (Eisenberg-Bord and Schuldiner, 2017). The nucleus controls mitochondrial gene expression and posttranslational modifications, the so-called anterograde signaling, and the mitochondria modulate nuclear gene expression and cellular protein activity through signal transport originating from the mitochondria, termed retrograde signaling. Additionally, in response to mitochondrial stress, the mitochondria can also send extracellular cues known as mitokines to affect nuclear regulation and modulate cellular or organismal homeostasis. For example, a second messenger such as hydrogen peroxide can be utilized by the mitochondria for hypoxia-inducible factor 1 (HIF-1) and NRF2 signaling for adaptive responses (Quirós et al., 2016).

The signaling between the mitochondria and nucleus is crucial for the homeostasis of the intracellular community, especially in response to cellular stress. Tsou *et al*. demonstrated that moderate physical exercise activated NRF2-dependent mitochondrial biogenesis and improved Parkinson's disease symptoms in MPTP models (Tsou et al., 2015). Parkin is also shown to regulate the PGC-1α-mediated transcription of nuclear genes encoding for TFAM, TFB2M, and complex subunits, favoring mitochondrial biogenesis (Shin et al., 2011). The transcriptional activities and the expressions of *PINK1* and Parkin genes are positively regulated by NRF-1 in dopaminergic cell SH-SY5Y, which activate mitochondrial quality control through mitophagy (Lu et al., 2020). NRF2-dependent transcription of the hereditary PD gene *PINK1* has also been identified in helping to save oxidative-stress-induced cell death (Murata et al., 2015). Moreover, NRF2 plays a part in parkin-mediated mitophagy by regulating the expression of p62/SQSTM1, an adapter molecule for linking ubiquitinated cargo directly with the autophagosome (Yang et al., 2019b). The disruption of p62/SQSTM1-dependent mitophagy has been shown to be pathological to PD (Sanchez-Martin and Komatsu, 2018). Thus, multiple signaling components involved in transcriptional adaptations governing the mitochondria and nuclear communication should provide potential targets for salvaging damaged mitochondrial networks and possibly offer therapies for PD (Glaab and Schneider, 2015; Blaudin de The et al., 2016; Kelly et al., 2019).

## Mitochondria, Endoplasmic Reticulum and PD

The ER consists of the nuclear envelope and a reticulated interconnected network of tubules and sheets. These ER sheets are studded with ribosomes and provide the entryway for proteins into the secretory pathway (Salvador-Gallego et al., 2017). ER tubules are observed to move dynamically on microtubules and tether to membranes of other organelles but do not fuse. Communication between ER and mitochondria is essential for the eukaryotic cells to integrate cellular physiology under changing environments. Mitochondria form close physical contacts (15–50 nm) with a specialized domain on the ER membrane, known as the mitochondria-associated membrane (MAM) (Martinvalet, 2018). These contact sites between the two organelles are called the mitochondria–ER contacts (MERCs), and up to 5–20% of mitochondrial surface is distributed in opposition to the smooth or rough ER (Bartok et al., 2019). This association constitutes a key signaling hub to regulate several fundamental cellular processes including lipid metabolism, inflammation, Ca^2+^ signaling, cell survival, autophagy, intracellular motility of both organelles, and protein homeostasis through close physical contacts and protein tethers (Gomez-Suaga et al., 2018; Barodia et al., 2019). Mounting evidence indicates that perturbed ER–mitochondria signaling contributes to many neurodegenerative diseases, including PD.

### MERCs Tethers

**Figure 2:**
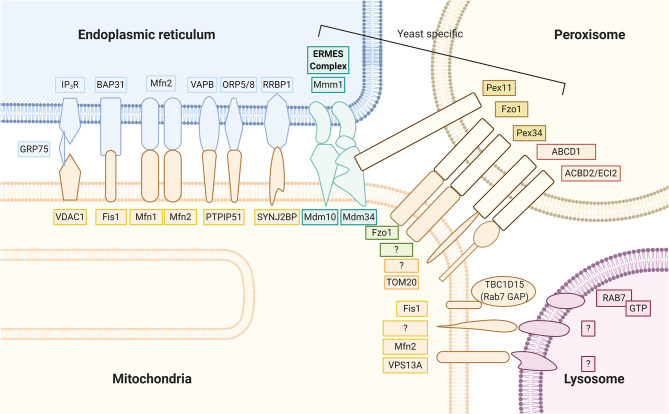
Mitochondria keep close contact with intracellular organelles within the cellular community in order to coordinate different intracellular functions. The MAM of the ER harbor at least seven proteinaceous tethers that bridge to the mitochondria, including (1) the ER Ca^2+^ channel IP3R and the MOM Ca^2+^ channel VDAC1 with the mitochondrial molecular chaperone GRP75 linked between; (2) the ER Bap31 with the mitochondrial Fis 1; (3) the ER Mfn2 with MOM Mfn1/2; (4) the integral ER protein VAPB to MOM protein PTPIP51; (5) the ER ORP5/8 also binds to mitochondrial protein PTPIP51; (6) the ER RRBP1 partners with MOM protein SYNJ2BP; (7) the yeast cell protein complexes containing Mdm12, Mdm34, Mdm10, and Mmm1 proteins that connect ER and mitochondria, called the ER–mitochondria encounter structure (ERMES). Peroxisomes partner with the mitochondria through (1) yeast Pex 11 to ERMES Mdm34; (2) yeast mitofusin homolog, Fzo1 on both peroxisome and MOM; (3) yeast Pex 34 on peroxisomes to unknown MOM partner; (4) mammalian ABCD1 on the peroxisomal membrane, whose loss of function causes X-linked adrenoleukodystrophy, is a peroxisome-mitochondria tether; (5) mammalian ACBD2/ECI isoform A with mitochondrial import receptor subunit TOM20. Mitochondrial contact with lysosomes was recently noted; however, exact tethers are unclear. Studies have clarified a major contact tethering promoter, lysosomal GTP-bound Rab7. Conversely, recruitment of cytosolic TBC1D15 (Rab7 GAP) to the mitochondria outer membrane via Fis1 leads to hydrolysis of GTP-bound Rab7. The resultant GDP-bound Rab7 dislodges from the lysosomal membrane and causes mitochondria and lysosome untethering. At the interface between the mitochondria and lysosome, studies have suggested MOM proteins VPS13A and Mfn2 to play functional roles.

Several proteinaceous tethers that bind the two organelles together with specific biological functions have recently been identified (Figure 2). Among these, one of the most well-known tethers that mediates Ca^2+^ transfer from the ER to mitochondria is the ternary binding complex, composed of the ER Ca^2+^ channel inositol 1,4,5-trisphosphate receptor (IP_3_R) and the major MOM Ca^2+^ channel voltage-dependent anion channel isoform 1 (VDAC1), with the mitochondrial molecular chaperone glucose-regulated protein 75 (GRP75) linked between (Leipnitz et al., 2018). Other tethering proteins include the interaction of the ER B-cell receptor-associated protein 31 (Bap31) with the mitochondrial Fis1 protein, which is associated with promotion of apoptosis. Another proposed tethering complex is the homotypic and heterotypic link of ER Mfn2 with MOM Mfn1/2, the functioning of which remains unclear (Filadi et al., 2018). As commonly proposed, groups including Naon et al. confirmed Mfn2 as a bona fide ER–mitochondria tether whose ablation decreases interorganellar juxtaposition and communication (Naon et al., 2016; Basso et al., 2018); meanwhile, groups including Leal et al. and Filadi et al. revealed that Mfn2 knockdown showed a contradictory increase in the ER–mitochondria association (Filadi et al., 2015; Leal et al., 2016). Another important tether is the binding of integral ER protein vesicle-associated membrane protein associated protein B (VAPB) to MOM protein tyrosine phosphatase-interacting protein 51 (PTPIP51), which is associated with Ca^2+^ regulation and autophagy (Gomez-Suaga et al., 2017b). PTPIP51 also binds to other mitochondrial proteins localized in the MAM, the oxysterol-binding protein-related proteins (ORP5/8) (Lee and Min, 2018). The phosphofurin acidic cluster sorting protein 2 (PACS2), a key ER protein required for MAM assembly and activity, has also been suggested to be involved with ER–mitochondria association integrity, influence MAM lipid metabolic enzymes activity, and regulate localization of the ER chaperone calnexin and the Ca^2+^ channel transient receptor potential protein 2 (TRPP2) at the MAMs (Rodriguez-Arribas et al., 2017). In yeast cells, a structural protein complex that connects ER and mitochondria called the ER–mitochondria encounter structure (ERMES), has been reported to contain Mdm12, Mdm34, Mdm10, and Mmm1 proteins, which allow for efficient lipid transport (Lee and Min, 2018). Most ER–mitochondrial tethers are found on smooth ERs; however, Victoria Hung *et al*. identified an interesting MOM protein synaptojanin 2 binding protein (SYNJ2BP) that joins with ER partner ribosome-binding protein 1 (RRBP1), whose overexpression dramatically increased rough ER–mitochondria association (Hung et al., 2017).

### Mitochondria and ER Communication Regulating Calcium Transfer and Mitochondrial Dynamics

ER is the major intracellular Ca^2+^ store of cells, while mitochondria shape and decode cellular Ca^2+^ signals by taking up and then releasing Ca^2+^ ions at specific positions throughout the cell (Santo-Domingo and Demaurex, 2010). Ca^2+^ transfer from ER to mitochondria has an important function in regulating cellular health, since basal Ca^2+^ concentration is necessary to maintain mitochondrial ATP and protein production due to three TCA cycle dehydrogenases and FAD-glycerol phosphate dehydrogenase, which are activated by Ca^2+^ (Giorgi et al., 2018). The MERCs are hotspots for Ca^2+^ signaling and are enriched with Ca^2+^ channels such as the IP3R–GRP75–VDAC complex and IP3R-related chaperones (calnexim and calreticulum), the VAPB–PTPIP51 complex, the Mfn complex, while other regulators include the ER Ca^2+^ transport ATPase (SERCA2b) (McDonnell et al., 2019). Ca^2+^ freely passes MOM through the VDAC, while the main control of mitochondrial matrix Ca^2+^ concentration is through the MIM mitochondrial Ca^2+^ uniporter complex (Steffen and Koehler, 2018). However, in the situation of Ca^2+^ transfer blockage, ATP production decreases and autophagy is triggered (Tubbs and Rieusset, 2017). Furthermore, in prolonged Ca^2+^ overload, mitochondrial Ca^2+^ concentration surpasses a threshold, and mitochondrial permeability transition pore (mPTP) opens permanently, leading to dissipation of the Δψm and subsequent apoptosis (Krols et al., 2016). Thus, mitochondrial Ca^2+^ plays a dual role, and its homeostasis is intimately linked to both cell survival and death (Ilacqua et al., 2017).

Mitochondrial function can also be affected by ER; for example, MAM on ER influences mitochondrial dynamics including fission, trafficking, and mitophagy (Prudent and McBride, 2016). Friedman et al. noted that ER tubules wrap and constrict around mitochondria, which recruit Drp1 to these MERCs and promote MOM fission (Friedman et al., 2011). Later studies by Chakrabarti et al. showed that actin polymerization through ER-bound inverted formin 2 (INF2) stimulated fission of both MIM and MOM via increased mitochondrial matrix Ca^2+^ and Drp1 recruitment, respectively (Chakrabarti et al., 2018). Other mitochondrial fission-involved proteins that recruit Drp1 including mitochondria fission factor (Mff), Fis1, and syntaxin 17 are also localized to the MERCs (Arasaki et al., 2015). For cells under high energy demand such as neurons, being the primary generators of ATP, the mitochondria are dynamically transported intracellularly, presumably for appropriate distribution to cellular regions of high metabolic demand and elevated intracellular calcium. ER has been found to play a role in mitochondrial trafficking. Mitochondrial trafficking along microtubules relies on the Miro GTPases, which localize to MOM and form complexes with the TRAK adaptors and dynein/kinesin motors (Murley et al., 2013). These molecular machineries are tightly regulated by Ca^2+^ sensors, and increased Ca^2+^ levels have been shown to slow down or stop mitochondrial motility, which is reversible by the return of Ca^2+^ concentrations to a normal range. Souvik et al. have shown Miro clusters to increase MERCs, and other reports also have shown Miro regulation of the IP3R–GRP75–VDAC complexes for Ca^2+^ transfer (Lee et al., 2018; Modi et al., 2019). Under pathological conditions, high levels of Ca^2+^ in mitochondria have been shown to reduce both ER and mitochondrial trafficking and tighten the ER–mitochondria alignment, causing mitochondrial arrest (Grimm, 2012; Lee et al., 2016). This arrest will enhance Ca^2+^ transfer from ER and induce mitochondrial fragmentation in order to remove damaged organelles through mitophagy, initiated with membranous structures contributed by nearby MERCs (Jeyaraju et al., 2009; Hom et al., 2010; Steffen and Koehler, 2018). The VAPB–PTPIP51 complex regulate autophagy by tightening the MERCs and affecting Ca^2+^ transfer. Knockdown of either VAPB or PTPIP51 loosens the MERCs and stimulates autophagosome formation, whereas overexpression of either inhibits autophagosome formation (Gomez-Suaga et al., 2017a). Hamasaki et al. demonstrated in mammalian cells that pre-autophagosome/autophagosome marker ATG14 (also known as ATG14L) relocalizes to the MERCs after starvation, and the autophagosome-formation marker ATG5 also localizes at the MERCs until autophagosome formation is complete (Hamasaki et al., 2013). They also showed that disruption of the ER–mitochondria contact site prevents the formation of ATG14 puncta, emphasizing the importance of MERCs during early stages of autophagy (Dikic and Elazar, 2018). The implication of MERCs in mitophagy includes the ubiquitination-mediated PINK1 and parkin-related pathway and the mitophagy receptor-mediated pathway through the FUN14 domain containing 1 (FUNDC1). Wu et al. demonstrated that, under hypoxic conditions, FUNDC1 initiates mitophagy at the MERCs via interaction with calnexin and recruitment of upstream autophagy protein Unc-51-like kinase 1 (ULK1) (Wu et al., 2014). Wu et al. also observed FUNDC1 mediation of mitochondrial fission in hypoxia by binding to Drp1 (Wu et al., 2016). An interesting soluble N-ethylmaleimide-sensitive factor attachment protein receptor (SNARE) protein syntaxin 17 has been shown to play multiple roles between the mitochondria, ER, Golgi, and lysosome. Syntaxin 17 localizes at the MERCs to promote mitochondrial fission by recruitment of Drp1 (Arasaki et al., 2015). During starvation, syntaxin 17 translocalizes to MERCs and binds and recruits ATG14 to initiate the formation of phagophores (Hamasaki et al., 2013; Wang et al., 2019). In the late stage of autophagy, syntaxin 17 present on autophagosomes, mediating the fusion of autophagosomes with lysosomes (Arasaki et al., 2017). Xian et al. identified the syntaxin 17–Fis1 interaction control of syntaxin 17 shuttling between mitochondria and ER and that Fis1 loss results in aberrant syntaxin 17 accumulation in the mitochondria, further triggering mitophagy (Xian et al., 2019). Another response noted concerning MERCs is the unfolded protein response (UPR)-ER, which is triggered by ER stress and accumulation of misfolded proteins aiming to maintain cellular proteostasis (Hetz and Papa, 2018; Kopp et al., 2019). Many UPR-ER-related chaperones are found in the MAM, and the disruption of ER–mitochondrial signaling by the array of tethering proteins can induce UPR-ER including PACS2, Mfn2, and VAPB (Paillusson et al., 2016; Chu et al., 2019).

### The Role of MERCs in PD

The role of MERCs in PD involves Ca^2+^ regulation, proteostasis, and several familial PD-related proteins (Kazlauskaite and Muqit, 2015; Basso et al., 2018). The α-syn is a well-known PD pathogenic protein and has been observed in membrane compartments of synaptic vesicles, mitochondria, and ER in neurons. α-Syn is enriched in the MAMs, and Cali et al. have reported that overexpression of either wild-type or familial mutant α-syn decreases ER–mitochondria communication, damages Ca^2+^ homeostasis, induces mitochondrial fragmentation, and augments autophagy (Cali et al., 2012). Supporting this, Paillusson *et al*. used structured illumination and electron microscopy to quantify MERCs and revealed that α-syn binds to VAPB and that overexpression of wild-type or mutant α-syn disrupts the VAPB-PTPIP51 tethers to loosen ER–mitochondria associations and further affect Ca^2+^ signaling (Paillusson et al., 2017). The autosomal recessive PD risk genes *PINK1* and *parkin* help to maintain mitochondrial health through several mitochondrial quality control mechanisms: the turnover of MOM proteins by the proteasome, the generation of mitochondrial-derived vesicles, and whole-organellar degradation by mitophagy. More recently, PINK1 and parkin have been found to localize at MERCs to modulate organellar crosstalk. At MERCs, the PINK1/parkin-dependent mitophagy pathway is involved in disturbed Ca^2+^ transferring, mitochondrial fragmentation, trapped mitochondrial movement, loosening ER/mitochondrial contact, recruitment of autophagic machinery to dysfunctional mitochondria, and acquirement of membrane components from the ER for autophagosome membrane formation (Amadoro et al., 2014; Barazzuol et al., 2020). The importance of mitochondria–ER association in the initiation stage of autophagosome has been revealed by Yang et al., such that parkin-mediated mitophagy recruits autophagosome precursors to the MERCs at damaged mitochondria and LC3-marked autophagic structures emerge from the ER–mitochondria contact sites (Yang and Yang, 2013). Gelmetti et al. noted PINK1 and parkin to relocalize at MERCs, where autophagosome originate, and that *PINK1* silencing impaired pro-autophagic protein enrichment at MERCs (Gelmetti et al., 2017). At the start of PINK1/parkin-dependent mitophagy, MERCs-localized Mfn2 is phosphorylated by PINK1, and the phosphorylated Mfn2 recruits parkin, which ubiquitinates Mfn2. Accumulation of ubiquitinated Mfn2 on the MOM acts as a signal to mark damaged mitochondria and initiates mitophagy (Bockler and Westermann, 2014). After mitophagy initiation, mitochondria–ER contacts are dissociated, as PINK1/parkin catalyzes Mfn2 to dissemble Mfn2 from the MOM (Basso et al., 2018). The loosening of ER/mitochondrial contact in mitophagy pathways has been shown to increase the rate of mitochondrial degradation, and McLelland et al. demonstrated that MERCs tethers suppress mitophagy (McLelland et al., 2018). Parkin has also been shown to accumulate at MERCs to modulate ER–mitochondrial crosstalk, and overexpression or silencing of *parkin* is correlated to Ca^2+^ dyshomeostasis (Cali et al., 2013; Gautier et al., 2016). In *Drosophila* models of PD, mutations in *parkin* and *PINK1* induce ER stress through activating PERK (Celardo et al., 2016). Another PD recessive risk gene is *PARK7*, encoding the DJ-1 protein that may be important in both sporadic and familial PD (Blackinton et al., 2009). In most contexts, DJ-1 plays multiple protective roles in cells as redox sensors, antioxidants, chaperone with protease activity, and transcription regulator. Liu *et al*. reported that DJ-1 physically interacts with and is an essential component of the IP3R3–Grp75–VDAC1 complexes at MAM. Loss of DJ-1 disrupted the IP3R3–Grp75–VDAC1 complex and led to loosened ER–mitochondria association and disturbed function of MAM and mitochondria in neuronal cells and *in vivo* (Liu et al., 2019b). Although DJ-1 is often suggested to be protective, Yang et al. noted that deficiency of DJ-1 ameliorates death in the context of acute ER stress *in vitro* and *in vivo*. By contrast, overexpression of wild-type and PD-associated pathogenic DJ-1 mutant forms of PARK7 L166P enhance ER-stress-induced neuronal death by regulating activating transcription factor 4 (ATF4) transcription and translation (Yang et al., 2019a).

### The Golgi Apparatus, ER, Mitochondria, and PD

Working closely with ER, the main function of the Golgi apparatus is to modify and package proteins and carbohydrates into membrane-bound vesicles and dispatch these cargoes for exportation. The Golgi apparatus and mitochondria also communicate with each other by physical interaction. The existence of Ca^2+^ gradients from the Golgi apparatus to mitochondria has been discovered. Structurally, apposition of the Golgi apparatus and mitochondria has been demonstrated by microscopy techniques; however, the molecular features of this interaction in PD pathophysiology remain poorly understood (Valm et al., 2017). The involvement of ER/Golgi in the α-syn pathology has been further elucidated recently by Paiva *et al*. who reported that PD pathogenic A30P mutant α-syn causes alteration of Golgi morphology and increases the susceptibility of dopaminergic neurons to ER stress (Paiva et al., 2018). It has also been observed that overexpression of PD pathogenic A53T mutant α-syn delays ER–Golgi transport by up to 50% via inhibiting ER/Golgi vesicle fusion-related SNARE complex assembly (Thayanidhi et al., 2010). The SNARE protein, syntaxin 17, assists in vesicle fusion and is also a receptor at the ER membrane that mediates trafficking between the ER and ER–Golgi intermediate compartment (ERGIC) as well as localizes to MERCs for mitochondrial fission (McLelland et al., 2016; Sugo et al., 2018). The interesting interorganelle communication of MDVs are a means of vesicular transport of selectively incorporated protein cargoes from the mitochondria to other subcellular compartments, mainly lysosomes or peroxisomes, thereby transferring mitochondrial proteins to these organelles (Sugiura et al., 2014; Giacomello et al., 2020). The finding that syntaxin 17 participates in PINK1/parkin-dependent MDVs fusion within the endolysosomal compartments suggests a role of ER/Golgi interorganelle communication in mitochondrial dynamic control (Muppirala et al., 2011; Arasaki et al., 2015). Another participator of MDVs transport is the vacuolar protein sorting-associated protein 35 (VPS35), a gene product of autosomal dominant late-onset PD gene *VSP35*, which is involved in retrograde transport of proteins from endosomes to the trans-Golgi network (Yun et al., 2017). This retromer complex is also found to regulate MDVs cargo transport from the mitochondria to peroxisomes (Grunewald et al., 2019). More recently, there has been the discovery of Golgi apparatus involvement in late-stage mitochondrial fission. Nagashima et al. documented that microdomains of phosphatidylinositol 4-phosphate [PI(4)P] on trans-Golgi network vesicles were recruited to MERCs during late stage of mitochondrial division, the loss of which impeded fission, causing extended mitochondrial constriction sites with hyperfused and enlarged mega-mitochondria. This indicated that these Golgi-derived vesicles may drive the final events of mitochondrial division downstream of Drp1, leading to mitochondrial scission (Nagashima et al., 2020).

## Mitochondria, Peroxisomes, and PD

Peroxisomes are ubiquitous, single membraned cellular organelles that do not contain DNA or RNA. They perform important roles in biosynthesis and signal transduction, including phospholipid biosynthesis, fatty acid α- and β-oxidation, bile acid and docosahexaenoic acid synthesis, glyoxylate metabolism, amino acid catabolism, polyamine oxidation, the metabolism of reactive oxygen and nitrogen species, inflammation, and innate immunity (Islinger et al., 2018). Peroxisomes are dynamic, interconnected, and actively contribute to signaling, developmental decisions, aging, and defense against pathogens. To exert these activities, peroxisomes must interact both functionally and physically with other cellular organelles. Numbers, morphology, and activity are modulated to adapt to diverse environments in different tissues, organs, and nutritional states (Farre et al., 2019).

The crosstalk between mitochondria and peroxisomes is essential for several metabolic processes including redox-(ROS scavenging), lipid-(β-oxidation), inflammatory-, and innate immune-(antiviral responses) signaling networks (Fransen et al., 2017). The efficient exchange of molecules between mitochondria and peroxisomes are poorly understood but are likely to involve shuttle mechanisms such as the carnitine system, membrane pores, vesicle transport, and contact sites, which have been shown to localize at MERCs (Giacomello et al., 2020). The existence of peroxisome–mitochondria contact sites was confirmed by bimolecular fluorescence complementation (BiFC), and Shai et al. discovered yeast peroxisome–mitochondria contact site tether proteins: Fzo1 (yeast mitofusin protein) and peroxin (Pex) 34 (Shai et al., 2018; Farre et al., 2019). A handful of other tethers between peroxisomes and mitochondria have also been discovered, including peroxisome protein Pex11 to mitochondrial Mdm34, an ERMES component in yeast; mammalian peroxisome ATP-binding cassette subfamily D member 1 (ABCD1); and enoyl-CoA delta isomerase 2 (ECI2, also known as ACBD2) isoform A in mammals, which is linked to translocase of outer mitochondrial membrane 20 (TOM20) (Fan et al., 2016; Giacomello et al., 2020) (Figure 2). The passage of molecules through the peroxisome membrane is achieved through nonselective channels such as mammalian peroxisomal membrane protein 2 (PXMP2). Solutes with molecular masses smaller than 300 Da have free transmembrane movement, while larger molecules such as fatty acids, acetyl-CoA, and ATP pass through specific transporter proteins (Theodoulou et al., 2013). The integrity and stability of peroxisomes are important for the maintenance of normal mitochondrial function. Peroxisome dysfunction seriously affects mitochondrial metabolism, morphological stability, and biosynthesis, which directly or indirectly lead to rare genetic diseases, such as X-linked adrenoleukodystrophy (X-ALD), acatalasemia, and Zellweger syndrome, and age-related disorders such as PD (Muntau et al., 2000; Pascual-Ahuir et al., 2017; Uzor et al., 2020).

### A Role for Mitochondria in Peroxisome Biogenesis

A characteristic feature of peroxisomes is that they proliferate, change internal enzymes, and dissipate in response to external cues; the subsequent degradation, once they are excessive or non-functional, is through selective autophagy called pexophagy (Cho et al., 2018). The biogenesis of peroxisomes implicates important peroxisome biogenesis proteins known as peroxins, which modulate import of peroxisome matrix proteins and help target peroxisomal membrane proteins to the peroxisome membrane. The proteins destined for peroxisomes possess peroxisome targeting signals (PTS1 and PTS2) and are translated on free cytosolic ribosomes and then transported directly or indirectly into peroxisomes as completed polypeptide chains (Kim and Hettema, 2015). Two main models of peroxisome biogenesis in mammals have been proposed: one is by growth and division of existing peroxisomes (South et al., 2000); the other is *de novo* peroxisome biogenesis by the insertion of peroxisome membrane proteins into a specific region of ER or mitochondria membrane, which later buds to form pre-peroxisomal vesicles. These pre-peroxisomal vesicles containing different subsets of peroxisomal membrane proteins then fuse and mature into peroxisomes (Farre et al., 2019). In either peroxisomal biogenesis or growth, there are two proposed routes for peroxisomal membrane protein insertion into peroxisomes: one is through direct insertion of peroxisomal membrane proteins into membranes of pre-existing peroxisomes, and the other involves indirect trafficking of peroxisomal membrane proteins via ER/mitochondria followed by their subsequent sorting into peroxisomes (Agrawal and Subramani, 2016). In support of mitochondrial participation in peroxisome biogenesis, experiments to observe *de novo* peroxisome biogenesis were performed on human fibroblasts lacking peroxisome, revealing that integral peroxisomal membrane proteins Pex3, Pex12, Pex13, Pex14, Pex26, PMP34, and ALDP were imported into mitochondria at the beginning of peroxisome biogenesis (Halbach et al., 2006; Kim et al., 2006). These mitochondrial-derived pre-peroxisomal vesicles then fuse with ER-derived pre-peroxisomal vesicles to form newly born peroxisome, as reported by McBride *et al*. who used a human fibroblast cell line lacking Pex3 obtained from a patient with Zellweger syndrome (Sugiura et al., 2017). The fusion of mitochondria-derived pre-peroxisomal vesicles in peroxisomal formation allows transfer of functional proteins, which may be a reason for the similar functions of the peroxisomes and mitochondria (Schrader and Pellegrini, 2017).

### Brotherly Traits Between Peroxisomes and Mitochondria

Consistency amid diversity is observed between mitochondria and peroxisomes (Chipuk and Luna-Vargas, 2017). The two share biogenesis transcriptional programs, which are both triggered by PPAR-γ and its coactivator PGC1-α, including genes involved in peroxisomal β-oxidation. In addition, peroxisomal and mitochondrial membranes share the same division machinery, including Drp1. While mitochondria have Fis1 and MFF as Drp1 receptors, peroxisomes have also their own specific Drp1 receptors, Pex11 (Otera et al., 2013; Shai et al., 2016). However, although peroxisomes and mitochondria share a dynamic nature in fission, trafficking, and degradation, unlike mitochondria, mature peroxisomes cannot fuse with one another (Schrader and Pellegrini, 2017). The localization of proapoptotic regulator protein BCL2-antagonist/killer (BAK) has been found in both MOM and peroxisome membranes, modulating membrane permeability and peroxisomal enzyme deficiency (Chipuk and Luna-Vargas, 2017; Hosoi et al., 2017).

Another well-known example of cooperation between these two organelles is the β-oxidation of fatty acids. Mitochondria and peroxisomes each own a distinct set of substrate-specific enzymes with peroxisomes only able to shorten, but not completely degrade, the fatty acid chains; while mitochondria are able to β-oxidize down to H_2_O and CO_2_ (Poirier et al., 2006). Thus, dietary and very long chain fatty acids target peroxisomes for β-oxidation, and the medium chain fatty acids metabolites as well as acetyl-CoA are guided to the mitochondria where further oxidation and ATP production in the TCA cycle take place (Reddy and Hashimoto, 2001).

Evidence has shown that mitochondria and peroxisomes are sophisticated redox signaling hubs, and most of the cellular redox reactions occur within mitochondria, peroxisomes, and ER, which are the main generators of H_2_O_2_ and other ROS (Lismont et al., 2015). Redox-regulatory enzymes are thought to assemble at a “redox triangle” formed by the three organelles, assembling “redoxosomes” that sense ROS accumulations and redox imbalances (Yoboue et al., 2018). Each organelle harbor their own antioxidative system (Fransen et al., 2012, 2017).

### Importance of Peroxisomal Health on Mitochondrial Integrity

Normal peroxisomal function is crucial for maintaining the health of the mitochondrial network. Multiple studies have shown deficiencies in peroxisome to damage mitochondrial integrity including morphology, mitochondrial proteostasis, redox balance, mitochondrial biogenesis, and even leading to cell death (Walton and Pizzitelli, 2012; Lismont et al., 2015; Schrader et al., 2015; Cipolla and Lodhi, 2017). Supporting this, increased mitochondrial protein oxidative damage and impairment of mitochondrial OXPHOS was observed by Lopez et al. in the spinal cord of mice with inactivated ABCD1, a peroxisome very-long-chain-fatty-acid transporter causative for X-ALD. The very long chain fatty acid substrate accumulates in the cytosol due to hindered import into the peroxisome for degradation and leads to progressive demyelination/neurodegeneration in the central nervous system (Lopez-Erauskin et al., 2013). Wang et al. demonstrated that excessive ROS production by peroxisomes led to mitochondria-induced cell death in Pex19p-deficient human fibroblasts and that this process may be countered by targeted overexpression of select antioxidant enzymes: peroxisomal glutathione S-transferase Kappa 1, SOD1, and mitochondrial catalase (Wang et al., 2013). Similarly, Peeters et al. demonstrated disruption of peroxisome biogenesis in hepatocyte-selective Pex5 knockout mice to damage MIM, deplete mtDNA, reduce or incomplete respiratory chain complexes I, III, and V, increase oxidative stress, increase mitochondrial membranes permeability and fluidity, and increase mitochondrial biogenesis (Peeters et al., 2015; Tanaka et al., 2019). Both peroxisome and mitochondrial dysfunction and dysregulated ROS balance is important in the occurrence of age-related disease (Cipolla and Lodhi, 2017). Several reports support that peroxisomal function progressively declines during aging and that, in cultured human cells catalase, is observed to be increasingly excluded from peroxisomes after repeated cell passage, thereby impeding the breakdown of H_2_O_2_. Concurrently, old cells accumulate old peroxisomes, increasing the ROS burden and ultimately may accelerate aging (Giordano and Terlecky, 2012; Pascual-Ahuir et al., 2017). Koepke et al. demonstrated that altering the catalase peroxisome targeting signal to the more effective serine–lysine–leucine (SKL) sequence results in a catalase molecule that more strongly interacts with its receptor and is more efficiently imported in both *in vitro* and *in vivo* assays. The catalase-SKL stably expressed in cells was able to repolarize mitochondria and reduce the number of senescent cells in a model of late-passage human fibroblast cell cultures, which may provide a potential strategy for rejuvenation (Koepke et al., 2007). Nell et al. showed genetically engineered derivative of the peroxisomal antioxidant enzyme catalase CAT-SKL usage to reduce neuroinflammation and long-term reference memory deficits induced by beta-amyloid in the mature rat brain (Nell et al., 2017).

### Peroxisomes and Mitochondria Relationship With PD

Peroxisomes have shown interdependency with mitochondria, and experiments have been conducted to investigate possible connections between peroxisomes and PD (Lazarou et al., 2012; Uzor et al., 2020). Lazarou et al. reported PD-related PINK1 and parkin ectopic localization to peroxisomes where they initiated pexophagy (Lazarou et al., 2012). Potential effects of peroxisomal dysfunction on α-syn-related pathogenesis were demonstrated on inactivated Pex2–/–, Pex5–/–, and Pex13–/– mouse models presenting increased α-syn oligomerization and deposition in cytoplasmic inclusions. Yakunin et al. further showed that α-syn abnormalities correlate with the altered lipid metabolism and specifically, with accumulation of long chain, n-6 polyunsaturated fatty acids, which occurs in peroxisome biogenesis dysfunctional models (Yakunin et al., 2010). These data demonstrate a role of peroxisomes in the prevention of α-syn aggregation, a pathological hallmark of PD. The mitochondria are integrated in the cellular endolysosomal system via MDVs, which transfers mitochondrial proteins and lipids by fusing with peroxisomes, endosomes, and lysosomes. The late-onset familial PD gene, *VPS35*, encodes a key component of the retromer complex for cellular protein trafficking, which also participates in generation and intracellular trafficking of MDVs to lysosomes (Braschi et al., 2010). Taken together, these findings support peroxisome participation in mitochondrial morphology dynamics, PINK1/parkin autophagic pathways, and cooperation with the mitochondria in shared stress responses such as ROS balancing in the pathogenesis of PD with aging.

## Mitochondria and Lysosomes and PD

Quality control of damaged mitochondria conducted by lysosomes is essential for maintaining normal mitochondrial functions. Lysosomes are single membrane-enclosed organelles that govern terminal degradation and act as sopisticated signaling centers of growth, division, and differentiation (Lim and Zoncu, 2016). They contain an array of ~50 acid hydrolases capable of breaking down all types of biological polymers—proteins, nucleic acids, carbohydrates, and lipids (Lawrence and Zoncu, 2019). Lysosomal hydrolases are activated under an acidic enviroment and rely upon vacuolar ATPase (V-ATPase) pumps in the lysosomal membrane to actively transfer protons into the lysosome, maintaning a pH of ~5. With the participation of hydrolases, lysosomes digest large molecules through autophagy and pass the fragments on to other parts of the cell for recycling. As the degradative endpoint for intracellular and exogenous biomacromolecules, these cellular quality controllers continuously fuse and fission with each other as well as other organelles, including late endosomes, phagosomes, and autophagosomes for autolysosome formation (Wong and Cuervo, 2010). The chemistry between mitochondria and lysosomes facilitate sensing the availability of nutrients and energy, coordinating anabolic and catabolic processes, as well as coping with cellular stress in autophagy, proliferation, and cell death (Raimundo et al., 2016). The means of communication between the two organelles include signaling pathways, MDVs, fusion with damaged mitochondria for degradation, and “kiss and go” membrane contacts dynamically formed with healthy mitochondria. The formation of these physical contact sites are promoted by lysosomal active GTP-bound Rab7 and are dissociated by deactivated Rab7 GTP via Rab7 GTPase-activating protein TBC1D15 (Rab7 GAP); however, the tethers that bridge the contact sites in mammals are as yet unclear (Wong et al., 2018) (Figure 2).

### Mitochondria Quality Control and Lysosomes

The most well-known interaction between mitochondria and lysosomes is in the selective autophagy of mitochondria, called mitophagy. This housekeeping mechanism of the mitochondrial network is critical for mantaining efficient working organelles within the cell and prevent excessive production of ROS from malfunctioning organelles. Autophagy is a process in which lysosomes degrade unneeded or damaged large molecules in cells through segregation in a double-membraned vesicle and lysosomal fusion with the vesicle enabling hydrolase function. There are three types of autophagy, which include microautophagy, chaperone-mediated autophagy, and the most common macroautophagy. Henceforth, we refer to macroautophagy as autophagy, which is also the process involved in mitophagy. Steps of autophagy include initiation, nucleation, elongation, lysosomal fusion, and degradation. The initiation stage involves activating ULK1 complex, which phosphorylates the class III phosphatidylinositol 3-kinase complex I (PI3KC3-C1) (Feng et al., 2019). The PI3KC3-C1 assists phosphatidylinositol 3-phosphate [PI(3)P] production at the isolation membrane of the ER, from which transient double-membraned phagophores are formed (Hurley and Young, 2017). Downstream proteins and complexes work to enhance autophagy-related proteins ATG8-family proteins (ATG8s) binding to the phosphatidylethanolamine (PE) on the membrane. ATG8s [including the microtubule-associated protein light chain 3 (LC3) proteins] assist in the nucleation process, which recruits LC3-interacting-region (LIR)-motif-bearing autophagy factors, and selectively sequester specifically tagged cargo via LIR cargo receptors (Wirth et al., 2019). While ATG8s also facilitate elongation and closure of the phagophore membrane to form the autophagosome, the insertion of lipidated-ATG8s to the autophagosome membrane drives autophagosome maturation (Johansen and Lamark, 2019). Thus, the binding of lipidated ATG8s family member LC3-II to the autophagic membrane is suggested to be a signature characteristic of autophagic activation (Wild et al., 2014). The elongation of autophagosomal membranes is facilitated by ATG9-containing vesicles bringing lipid bilayers from the plasma membrane, the mitochondria, recycling endosomes, and the Golgi complex. Autophagosomes are trafficked to and fuse with a lysosome, forming an autolysosome, and trapped cargo is subsequently degraded (Klionsky et al., 2014). This, therefore, is the well-known incinerating function of lysosomes in cellular degradation; the elimination of perturbed organelles via selective autophagy in the case of mitochondria is mitophagy (Raimundo et al., 2016). The warning signal for damaged mitochondria is membrane depolarization, after which MOM proteins are ubiquitinated, mitophagy receptors are recruited, autophagosomes encircle the damaged mitochondria, and finally fuse to lysosomes for degradation (Palikaras et al., 2018; Liu et al., 2019a). A second type of mitochondrial quality control involving lysosomes is the excision of MDVs from the mitochondria, which fuse with lysosomes to degrade damaged parts of mitochondria (Sugiura et al., 2014). Both mitochondria and lysosomes are self-aware of their functional status and constantly relay organelle conditions and stresses to the rest of the cell (Tai et al., 2017). Lysosomes achieve this by retaining Ca^2+^, iron, cholestrol, or sphingomyelin within the lysosomal lumen (Audano et al., 2018). The mitochondria do this by mediating Ca^2+^ uptake, ROS signaling, slowing protein input, effluxing peptides, vesiclular signaling, and regulating mtDNA expression (Haynes et al., 2010; Lin and Haynes, 2016; Shpilka and Haynes, 2018). Apart from initiation, the termination of stress responses is especially important to prevent extreme reactions that could lead to irreversible damage. When stress signals elevate over a threshold, housekeeping mechanisms may initiate the removal of the damaged organelle (Palikaras et al., 2018).

### Functional Crosstalk Between Lysosomes and Mitochondria

Intimate functional connections between the mitochondria and lysosomes are demonstrated by investigating the interactions between these two organelles. First, the removal of entirely damaged mitochondria through mitophagy involves autolysosome formation. Second, MDVs released from the mitochondria in response to acute mitochondrial damage are targeted for digestion to lysosomes. MDVs harbor selectively chosen mitochondrial proteins and detach from the mitochondria independently of Drp1 but require proteins that affect mitochondrial dynamics, such as parkin and PINK1 (Sugiura et al., 2014). Third, damaged proteins and lipids are compartmentalized in a specific area of mitochondria called mitochondria-derived compartments (MDCs) and are released involving Drp1, the mitochondrial fission machinery, to be degraded in the lysosome (Hughes et al., 2016). MDCs differ from MDVs in protein composition and have only been detected under chronic conditions, such as aging. In addition, MDCs primarily function in selective cargo degradation, while MDVs also play a role in protein exchange. Lastly, direct physical contacts are formed between these two organelles via membrane contact sites, allowing for the exchange of lipids and metabolites between these compartments (Giacomello et al., 2020).

Dysfunctional mitochondria have an impact on lysosomes (Demers-Lamarche et al., 2016; Fernandez-Mosquera et al., 2019). For example, knockout of the mitochondrial apoptosis-inducible factor (AIFM1) required for the assembly of respiratory chains leads to lysosomal impairment, evidenced by enlargement of specific lysosomal vesicles that become non-acidic and lose their hydrolytic activity (Demers-Lamarche et al., 2016). In reaction to imminent danger and to chronic stress, the two organelles collaborate on the modulation of intracellular metabolic processes via two major opposing metabolic regulators: the AMP-dependent protein kinase (AMPK) and mechanistic target of rapamycin complex 1 (mTORC1) signaling (Rabanal-Ruiz and Korolchuk, 2018). AMPK is the regulator that activates key catabolic pathways for the generation of ATP and is activated in response to low AMP/ATP, such as in a starvation condition (Mihaylova and Shaw, 2011). Meanwhile, mTORC1 is the kinase that coordinates most anabolic pathways, promoting synthesis of proteins, cholestrol, and nucleotides for cell growth and proliferation (Fernandez-Mosquera et al., 2019). Studies have revealed lysosomal biogenesis to increase in acute mitochondrial stress but not in chronic mitochondrial stress. In response to acute mitochondrial stress, AMPK is activated, which increases fission of damaged mitochondria, induces autophagosome formation through ULK1 and ULK2, increases lysosomal biogenesis through transcription factor EB (TFEB) and microphthalmia-associated transcription factor (MITF), leading to eventual mitophagy and increased autophagic flux (Nezich et al., 2015; Carroll and Dunlop, 2017). Under chronic mitochondrial stress, AMPK signaling is shut down, and TFEB returns to basal levels (Fernandez-Mosquera et al., 2017). This suggests that under chronic mitochondrial stress, a cellular protective mechanism is activated to stop mitochondrial cleanup, and the cell chooses to live with inefficient mitochondrial rather than without (Fernandez-Mosquera et al., 2019). AMPK also plays an important part in Ca^2+^ release from the lyososome into the cytoplasm by increasing the channel mucolipin-1 (MCOLN1), a key Ca^2+^-conducting channel on the lysosomal membrane that is essential for lysosomal biogenesis and autophagy (Medina et al., 2015). Elevated ROS production caused by mitochondrial dysfunction triggers AMPK activation with subsequent MCOLN1 activity stimulation, increased lysosomal activity, and potentially enhancing cellular capability to turn over damaged mitochondria (Zhang et al., 2016).

### Genetic Evidence of Lysosome, Mitochondria, and PD Connections

Recent genetic studies have revealed that mutations in the glucocerebrosidase (GBA1) gene cause Gaucher disease (GD), the most common lysosomal storage disorder, and increase susceptibility to PD and α-syn pathology (Klein and Mazzulli, 2018). The lysosomal storage diseases are caused by loss-of-function variants in genes that encode lysosome-digesting enzymes, leading to lysosomal dysfunction and consequential intralysosomal buildup of undegraded substrates (so-called “storage”) (Li et al., 2019a). The participation of lysosomal dysfunction on PD pathogenesis was first suggested in the recessively inherited lysosomal storage disorder, GD (Klein and Mazzulli, 2018). GD patients harbor homozygous mutations in the GBA1 gene, which encodes the lysosomal hydroxylase β-glucocerebrosidase for degrading the lipid glucosylceramide into ceramide and glucose. Typical clinical presentations include hepatomegaly, pancytopenia, osteoporosis, and neurological impairment in olfactory, neuromuscular, and cognitive systems (Magalhaes et al., 2018). Recently, Sidransky et al. discovered that GD patients presented with symptoms of PD (Sidransky, 2005). Lewy body pathology with α-syn-positive inclusions were also found in cortical and brain stem autopsies of GD patients (Bembi et al., 2003; Hruska et al., 2006). Furthermore, epidemiology studies demonstrated that family members of GD patients carrying heterozygotic-mutated GBA1 gene have significantly higher incidence of parkinsonism (Riboldi and Di Fonzo, 2019). Later studies demonstrated that PD patients had increased incidence of GBA1 mutations in comparison with control, while PD patients with GBA1 mutations had an earlier onset age (Robak et al., 2017). The clearance of α-syn is primarily mediated through autophagic-lysosomal systems, in which aggregated forms are degraded through (macro)autophagy and soluble forms through chaperone-mediated autophagy (Webb et al., 2003; Martinez-Vicente and Vila, 2013). In chaperone-mediated autophagy, the KFERQ domain of α-syn is recognized by the heat shock cognate 71 kDa protein (Hsc70) chaperone that targets α-syn to the lysosome. Upon arrival at the lysosome membrane, the lysosome-associated membrane protein type 2A (LAMP2A) receptor assists in the docking and internalization of α-syn into the lysosome, where α-syn is degraded by hydrolases (Kaushik and Cuervo, 2018). Lysosomal dysfunction decreases lysosomal enzymatic function, impairing α-syn autophagic clearance, and increases α-syn misfolding leading to an increase in damaged α-syn accumulation (Sidransky and Lopez, 2012; Mehra et al., 2019). Alternatively, α-syn accumulation disrupts GCase trafficking to lysosomes and decreases GCase activity, which further exacerbates the vicious cycle of protein misfolding underlying GBA-associated PD Lewy body formation, thereby forming a bidirectional feedback loop between α-syn and lysosomes (Bras et al., 2008; Wong and Krainc, 2016). Recently, extracellular transfer of misfolded α-syn throughout the brain has been suggested to involve neuron to neuron prion-like propagation between neuroanatomically connected areas. Jakob et al. demonstrated in a 3D-matrix differentiated human neuroblastoma model that, following autophagic failure, α-syn aggregates accumulate within the cell and the lysosomal system releases partially degraded α-syn via exocytosis to be taken up by neighboring cells through endocytosis. They also revealed that α-syn was colocalized with the lysosomal system, both pre- and postsynaptically (Domert et al., 2016). These findings suggest the potential of lysosomal involvement in the processes of interneuron spreading of α-syn pathology observed in the Braak pathology staging (Steiner et al., 2018).

Strong mitochondria-lysosomal interactions have also been found in other PD-susceptible genes. For example, deletion of *PINK1* of MOM in mouse cortical neurons resulted in lysosomal dysfunction including defective lysosomal acidification, decreased lysosomal activity, and large cytoplasmic late-endosome-marker-positive vacuole formation. Meanwhile, further addition of antioxidants to these mitochondria-dysfunctional neurons exhibited improvement of decreased lysosomal activity (Demers-Lamarche et al., 2016; Gomez-Sanchez et al., 2016). To preserve the integrity of oxidative-stressed mitochondria, PINK1/parkin are noted to be involved in the rapid lysosomal targeting of oxidized mitochondrial proteins via MDVs, a process that is PINK1/parkin dependent, autophagy independent, and lysosome targeted (McLelland et al., 2014; Roberts et al., 2016). DJ-1, the gene product of autosomal recessive PD-related *DJ-1*, has been shown to decrease α-syn aggregation via the lysosomal system. Xu et al. demonstrated that *DJ-1* knockout/down repressed α-syn degradation through inhibiting chaperone-mediated autophagy by lysosomes (Xu et al., 2017). DJ-1 has also been shown to regulate the proteolytic machinery 20S proteasome under an oxidizing environment, which is involved in protein homeostasis and ubiquitin-independent autophagosome–lysosome fusion (Tanaka, 2009; Moscovitz et al., 2015; Kumar Deshmukh et al., 2019; Njomen and Tepe, 2019). The most frequent PD gene, *LRRK2*, interacts with many proteins in the endo-lysosomal system and is involved in the steps of lysosome formation, trafficking, and autophagosome formation (Hockey et al., 2015). Meanwhile, *LRRK2* mutations are noted to interfere with mitochondria fission factor DLP1, causing mitochondrial dynamic imbalance and disturbing mitochondrial quality control. These combined effects lead to the eventual accumulation of damaged mitochondria (Salašová et al., 2017; Singh et al., 2019). A recent study by Ysselstein et al. discovered that the inhibition of LRRK2 kinase activity results in increased glucocerebrosidase activity in DA neurons with either *LRRK2* or *GBA1* mutations. This increase in glucocerebrosidase activity partially rescues accumulation of oxidized dopamine and α-syn in PD patient neurons (Ysselstein et al., 2019).

The autophagic-lysosomal pathway is also implicated in some less common familial PD genes (Pitcairn et al., 2019). Grünewald et al. and Gusdon et al. demonstrated that depletion of the ATP13A2, the gene responsible for a form of autosomal recessive juvenile-onset parkinsonism, is associated with impaired lysosomal acidification, decreased autophagic flux, diminished lysosomal-mediated clearance of autophagosomes, mitochondrial fragmentation, increased ROS production, higher frequency of mtDNA lesions, and decreased mitochondrial turnover (Grunewald et al., 2012; Gusdon et al., 2012). Supporting this, Veen et al. showed that ATP13A2 is an important lysosomal polyamine exporter, and defective lysosomal polyamine export causes lysosome-dependent cell death (Estrada-Cuzcano et al., 2017; van Veen et al., 2020). The lysosomal protein, VPS35, a key component of the retromer complex for cellular protein trafficking, mediates MDVs trafficking between mitochondria and other cellular compartments and is suggested to be associated with rare familial PD (Olszewska et al., 2016; Yun et al., 2017). The pathogenic D620N PD mutation in *VPS35* was shown to disrupt endosomal protein trafficking, autophagosome formation, and cause mitochondrial fragmentation and respiratory chain defects (Follett et al., 2014; Zavodszky et al., 2014; Zhou et al., 2017). As reported, *VPS35* and PINK1/parkin interaction occurred in the formation of mitochondria-derived vesicles, in which overexpression of *VPS35* salvaged parkin mutant phenotypes (Malik et al., 2015). In addition, depletion of *VPS35* in mouse neurons reduced mitochondrial fusion protein Mfn2 stabilization, impeded mitochondrial fusion, and resulted in mitochondrial fragmentation. In the same study, Tang et al. demonstrated that deletion of the *VPS35* gene in mouse DA neurons caused neuronal loss and α-syn accumulation (Tang et al., 2015). The mechanisms of mutant *VPS35* involvement in mitochondrial fission was also noted in the interaction with mitochondrial fission factor DLP1, as *VPS35* regulate recycling of DLP1 complexes. Mutant *VPS35* (D620N)–DLP1 interaction was shown to cause excessive mitochondrial fission and neuronal death, which are enhanced in oxidative stress (Wang et al., 2016). Recently, over 41 genetic susceptibility loci have been associated with late-onset PD in the largest genome-wide association studies (GWAS) meta-analysis of PD to date (Chang et al., 2017). One identified gene is the human transmembrane protein 175 (TMEM175), encoding the lysosomal K^+^ channel transmembrane protein (Jinn et al., 2019). Deficiency of TMEM175 has been revealed to impair lysosomal acidification, causing mitochondrial dysfunction, influencing α-syn phosphorylation, and impairing autophagy (Jinn et al., 2017). Also noted through the GWAS study was the sterol regulatory element-binding transcription factor 1 (SREBF1) that links lipogenesis to PD (Do et al., 2011; Ivatt and Whitworth, 2014). SREBF1 is a transcriptional activator imperative for the regulation of lysosomal lipid, and cholesterol accumulation and knockdown of the SREBF1 have been shown to block the translocation of parkin to mitochondria, consequently decreasing mitophagy (Gan-Or et al., 2015; Redensek et al., 2017). These findings indicate a reciprocal functional relationship between the mitochondria and lysosomes, with defects in one impacting the function of the other (Plotegher and Duchen, 2017).

## Conclusion

A major hurdle in the development of neuroprotective therapies for PD is the limited comprehension of key molecular pathways and targets in the pathogenesis of the disease. The involvement of the energy and metabolic factory, mitochondria, has long been associated with PD progression. Recent identification of physical contact sites between the mitochondria and multiple intracellular organelles has provided critical insight into clarifying how normal mitochondrial functioning extends beyond the organelle itself, exhibiting a complex array of dynamic behaviors. These highlight the bidirectional crosstalk as well as intracellular alarms and protective systems maintained by mitochondrial communication within the intracellular community. The interorganelle communication network expands the scope of investigation: mitochondrial dysfunction in PD pathogenesis is no longer an isolated event but impacts the entire cellular community. Despite recent insights, we lack a clear understanding at the molecular level of how hindered mitochondrial interorganelle communication affects PD pathogenesis.

Mitochondrial damage in the pathology of PD involves several key characteristics: morphological changes, loss of Δψm, protein misfolding and accumulation, decreased ATP production, Ca^2+^ dyshomeostasis, ROS imbalance, mtDNA mutation, autophagy, apoptosis, and lipid oxidation. Research aimed at strategies to maintain effective and efficient interorganelle balance and communication networks will be necessary to develop treatments for this neurodegenerative disease in the future.

## Author Contributions

T-KL contributed to concept generation, data interpretation, graphic drawing, and drafting of the manuscript. K-JL contributed to concept generation, graphic drawing, and drafting of the manuscript. K-LL contributed to concept generation and drafting of the manuscript. C-WL contributed to concept generation, data interpretation, and approval of the article. S-DC contributed to concept generation, data interpretation, and drafting of the manuscript. Y-CC, P-WW, J-HC, and T-JW contributed to concept generation, data interpretation, and approval of the article. All authors contributed to the article and approved the submitted version.

## Conflict of Interest

The authors declare that the research was conducted in the absence of any commercial or financial relationships that could be construed as a potential conflict of interest.

## Abbreviations

ABCD1: ATP binding cassette (ABC) transporter 1
mtDNA: Mitochondrial DNA
ACBD2(ECI2): Acyl-coenzyme A-binding domain
mTERF: Mitochondrial transcription termination factor
ADP: Adenosine diphosphate
nDNA: Nucleus DNA
APAF1: Apoptotic peptidase-activating factor 1
NCLX: Na^+^/ Ca^2+^ exchanger
ATF4: Activating transcription factor
NRF1: Nuclear respiratory factor 1
ATP: Adenosine triphosphate
OPA1: Optic dominant atrophy
ATP13A2: ATPase type13A2
ORP5/8: Oxysterol-binding protein-related proteins 5 and 8
α-syn: α-synuclein
OXPHOS: Oxidative-phosphorylation
BAK: BCL2-antagonist/killer
PACS2: Phosphofurin acidic cluster sorting protein 2
Bap31: B-cell receptor associated protein 31
PD: Parkinson's disease
BiFC: Bimolecular fluorescence complementation
Pex: Peroxin
Ca^2+^: Calcium
PGC: Peroxisome proliferator-activated receptors (PPAR) γ coactivator
DLP1: Dynamin like protein 1
PI3KC3-C1: Class III phosphatidylinositol 3-kinase complex I
DJ-1: Daisuke-Junko-1
PINK1: Phosphatase and tensin homologue (PTEN)-induced putative kinase 1
Drp1: Dynamin-related GTPase protein 1
PI(3)P: PI3KC3-C1 assists phosphatidylinositol 3-phosphate
ER: Endoplasmic reticulum
POLRMT: Mitochondrial RNA polymerase
ERGIC: ER-Golgi intermediate compartment
PPARs: Peroxisome proliferator-activated receptors
ERMES: ER-mitochondria encounter structure
PTPIP51: Phosphatase-interacting protein 51
ERRs: Estrogen-related receptors
PTS1/2: Peroxisome targeting signals 1 and 2
ETC: Electric transport chain
PXMP2: Peroxisomal membrane protein 2
FBXO7: F-box only protein 7
Rab7 GAP: Rab7 GTPase-activating proteins
Fis 1: Mitochondrial fission 1 protein
ROS: Reactive oxygen species
FUNDC1: FUN14 domain containing 1
Redox: Reduction-oxidization
GABPα: GA-binding protein-α (also called NRF2)
RRBP1: Ribosome- binding protein 1
GRP75: Glucose-regulated protein 75
rRNA: Ribosomal RNAs
GWAS: Genome-wide association studies
SERCA2b: Sarcoplasmic/endoplasmic reticulum Ca^2+^ pump 2b
HIF-1: Hypoxia inducible factor 1
SNARE: Soluble N-ethylmaleimide-sensitivefactor attachment protein receptor
IMS: Intermembrane space
SOD: Superoxide dismutase
INF2: Inverted formin 2
SREBF1: sterol regulatory element-binding transcription factor 1
IP3R: Inositol 1,4,5 trisphosphate receptor
SYNJ2BP: Synaptojanin 2 binding protein
LC3: Microtubule-associated protein 1A/1B-light chain 3
TCA: Tricarboxylic acid
LIR: LC3-interacting region
TFAM: Transcription factor A
LRRK2: Leucine rich repeat Kinase 2
TFB1M/ TFB2M: Transcription specificity factors
MAM: Mitochondria-associated membranes
TMEM175: Transmembrane protein 175
MCU: Mitochondrial Ca2^+^ uniporter
TOM20: Translocase of outer mitochondrial membrane 20
Mdm: Mitochondrial distribution and morphology
tRNA: Transfer RNAs
MDVs: Mitochondrial derived vesicles
TRPP2: Transient receptor potential protein 2
MERCs: Mitochondria-ER contacts
TWAS: Transcriptome-wide association study
Mff: Mitochondria fission factor
Ub: Ubiquitin
Mfn1/2: Mitofusin 1 and 2
ULK1: Unc-51-like kinase 1
Miro1/2: Mitochondrial Rho (Miro) GTPases
UPR: Unfolded protein response
MIM: Mitochondrial inner membrane
VAPB: Vesicle-associated membrane protein associated protein B
Mmm1: Maintenance of mitochondrial morphology protein 1
V-ATPase: Vacuolar ATPase
ΔΨm: Mitochondrial membrane potential
VDACs: Voltage-dependent anion-selective channel proteins
MOM: Mitochondrial outer membrane
VPS35: Vacuolar protein sorting 35
mPTP: Mitochondrial permeability transition pore
X-ALD: X-linked adrenoleukodystrophy
MPTP: 1-methyl-4-phenyl-1,2,3,6-tetrahydropyridine.
